# Improvement of Co-Composting by a combined pretreatment Ozonation/Ultrasonic process in stabilization of raw activated sludge

**DOI:** 10.1038/s41598-020-58054-y

**Published:** 2020-01-23

**Authors:** Hamideh Dastpak, Hasan Pasalari, Ahmad Jonidi Jafari, Mitra Gholami, Mahdi Farzadkia

**Affiliations:** 1 Research Center for Environmental Health Technology, Iran University of Medical Sciences, Tehran, Iran; 2 Department of Environmental Health Engineering, School of Public Health, Iran University of Medical Sciences, Tehran, IR Iran

**Keywords:** Biological techniques, Environmental sciences

## Abstract

The enhancement of composting technology to stabilize sludge pretreated by ozonation and ultrasonic was tested for 35 days. Secondary sludge produced by biological process are characterized with endogenous residue and inert solid matter which inhibit fully degrade bacterial cell walls. The composting process was performed with sludge pretreated with ozonatian and ultrasonics and green waste in a ratio of 2:1. The composting characteristics was evaluated for different physico-chemical and microbiological parameters in five different reactors. A high degree of composting quality was achieved with respect to significant reduction in volatile solids (VS) (32%), total organic carbon (TOC) (35.0%), C/N ratio (23.74), total coliform (TC) (168) along with the substantial increase in availability of nutrients like N (1.2%) and P (8.77%). High removal efficiency of TC and Fecal Coliform (FC) were observed in composting results, where simultaneous ultrasonic and ozonation were considered as primary-stabilization process. Therefore, applying integrated ultrasonic/ozonation with composting system for sludge stabilization is potentially useful technology in sustainable land restoration practices to meet standards and produce soil conditioner.

## Introduction

Overproduction of sewage sludge (SS), as an inevitable byproduct from activated sludge process, has currently led to use of inappropriate disposal practices^1^. In most developing countries, the traditional disposal methods including landfilling and open-dumping are being applied^2^. However, the presence of organic matter and highly polluted contaminants such as hazardous materials (i.e. antibiotics, bacteriostat, and flocculants) and heavy metals along with arbitrary discharge into environment have caused great concern about sludge management^3,4^. Challenges associated with sewage sludge require to be addressed. Improper SS disposal methods could cause secondary pollution containing pathogenic microbes, heavy metals and organic micro-pollutants which induce plant toxicity and alter the metabolism of microorganism present in soil culture^5,6^. Therefore, SS needs to undergo additional stabilization to decompose organic matter and reduce heavy metals to a recommended extent. On industrial scale, landfilling and incineration are recognized to be two environmentally accepted means of sewage sludge disposal^7^. However, the main drawback of these methods is that landfilling and incineration eliminate and remove the organic matter which may be considered for good uses^2,8^. Hence, there is utmost need to select methodology ecologically sound, economically viable and socially acceptable^9^. Composting and vermicomposting are well-known as ecologically and economically sustainable technologies for converting biosolids into safe and stable compost, which can be applied for subsequent uses like agriculture^10,11^. Microorganisms present in composting process accelerate organic degradation under controlled conditions; thermophilic stage enables sanitization of wastes and elimination of toxic compounds^9^. During composting, objectionable odors disappear quickly and the population of pathogenic microorganism reduce considerably^8^. Resultant products of composting are used as fertilizer in land application^10^. However, biooxidation processes may be inhibited by difficulty in degradation of the structural compounds^7^. A variety of chemical and physical treatment techniques have been applied to enhance lysis and disintegration of sewage sludge, including thermal hydrolysis^12^, mechanical disintegration^13^, freezing^14^, ultrasonic process^15,16^ and ozonation^17,18^. One of the preferred technologies for sludge degradation is ozonation. Ozonation decomposes the contaminants and high molecular weight compounds present in the sludge to low molecular structure. In addition, ozone can be converted to oxidizing radicals (i.e., hydroxyl radicals) and react with organic and inorganic fractions of sludge^19,20^. It can also oxidize the refractory compounds and convert them to biodegradable low-molecular compounds^20^. Ultrasonic is currently recognized as highly effective, environmentally friendly and cost-effective pretreatment method in sewage sludge management^16,21^. This process produces extreme condition, so-called, “hot-spots” with high temperature and pressure (5000 K, 1000 atm)^22^. Ultrasonic can disrupt the sludge flocks and allow the intracellular organic matter to extract from microorganism. Ultrasonic attack to unbounded and bonded water and increase total solid required for composting process with least energy expenditure^23^. Ultrasonic brings about sludge reduction following solubolization organic matter and enhance sludge biodegradability. Recently, many researchers have incorporated ozonation for sludge disintegration and improve COD Solubilization^19,24–27^ found that ozonation could considerably disintegrate sludge and ozonated sludge had high solubilized COD, which improve the sludge digestion. Zhang *et al*.^28^ observed that polysaccharides and proteins concentrations in supernatants were increased considerably after ozonation from 4.46 ± 0.21 mg/L to 220.90 ± 24.87 mg/L and 6.26 ± 0.28 mg/L to 386.54 ± 32.15 mg/L, respectively. Cheng *et al*.^29^ reported that ultrasonic, as pretreatment for sludge stabilization decrease oxidation-reduction potential in aerobic reactors and improve the release of intracellular substances into environment, which consequently enhance biodegradability. Kavitha *et al*^23^ reported a 60% dryness and dewatering can be obtained using ultrasonic without heat treatment. In addition, limited studies have focused on sludge stabilization using a combined ultrasonic and ozonation (Pei *et al*.)^20,30,31^ reported 30% and 28.10% sludge solubilization after applying ultrasonics (150,000 kJ/kg TS) and ozonation (0.1 g O_3_ /g TS), respectively. To the best of our knowledge, there are paucity of data concerning application of simultaneous ultrasonic in low frequency and ozonation process for biostabilization SS with composting process. Furthermore, most studies mentioned above utilized these processes as final sludge treatments. Therefore, the present research was developed for the first time to examine the performance of ozonation and ultrasonics as pretreatment in variations of physico-chemical and microbiological parameters of sludge. Additional aim of present research was to examine the ability of co-composting process in stabilization of raw activated sludge pretreated by ultrasonic and ozonation separately and simultaneously were evaluated.

## Results and Discussion

### Physico-chemical and microbiological parameters of sludge

The effects of ozonation on physio-chemical and microbiological characteristics of sludge were examined in consumed dose concentration of 6 g/L. As presented in Table 1, most physico-chemical properties of ozonated sludge including pH (7.2), TOC (37.32%), VS (67%), TN (1.01%), and TP (2.80%) had less values compared to initial ones. The findings of this primary-stabilized method are confirmed by other studies^28,32^. After ozonation and disintegration of sludge, C, N, and P were placed in micro-solid phase which are good source for microorganisms functions. Many studies suggest that ozone attack microbial cells and finally release the intracellular content^18,33^. Total (450) and Fecal coliform (150) of raw sludge decreased considerably after applying ozone. The operating conditions for ultrasonic were selected based on pretests and literature review. As shown in Table 1, a considerable reduction was observed in pH (7.5), VS (67.18%), TOC (37.35%), and on the other hand TN (1.8%) was increased after that sludge was exposed to ultrasonic radiation and all findings are confirmed by^23,29^. The results indicated that ultrasonic pretreatment favors sludge disintegration and improve the biodegradability process. The ultrasonic process didn’t change considerably TC (920) and FC (750), as microbiological parameters associated with sludge. It is noteworthy that the causes of variation of other parameters need further research and considerations. The sludge characteristics after applying simultaneous ozone and ultrasonic for primary-stabilization of raw sludge show that these parameters are in average values between values of ozonation and ultrasonic alone, except VS (77.97%), TOC (43.31%), and C/N (39.37%) ratio which had higher values compared to separate application of ozone and ultrasonic.

**Table 1:** Initial physico-chemical and microbial properties of green waste, raw sludge provided from MWTT, ozonized, ultrasonic and combined ozonation/ultrasonics sludge.

Physico-chemical and Microbial characteristics	Green waste	Raw sludge	Ozonized Sludge	Ultrasonic Sludge	Combined Ozonized/ Ultrasonic sludge
pH	7.30 ± 0.05	7.59 ± 0.04	7.2 ± 0.1	7.5 ± 0.08	7.35 ± 0.03
TS^a^	30.59 ± 0.10	2.59 ± 0.18	18.10 ± 1.2	17.92 ± 0.92	17.05 ± 0.35
VS/TS^a^	79.11 ± 0.08	89.18 ± 0.15	67 ± 0.13	67.18 ± 0.05	77.97 ± 0.07
TOC^a^	33.95 ± 0.12	39.55 ± 0.23	37.32 ± 0.15	37.35 ± 0.17	43.31 ± 0.14
TN^a^	0.78 ± 0.02	1.5 ± 0.03	1.05 ± 0.03	1.8 ± 0.08	1.1 ± 0.10
C:N Ratio	43.52 ± 0.37	26.37 ± 0.06	35.44 ± 0.14	20.73 ± 0.05	39.37 ± 0.01
TP^a^	8.5 ± 0.06	9.5 ± 0.10	2.8 ± 0.02	2.9 ± 0.03	2.5 ± 0.01
MC^a^	69.41 ± 0.69	97.50 ± 050	81.90 ± 0.75	82.08 ± 0.78	83.03 ± 0.12
TC^b^	400	1100	450	920	860
FC^b^	50	805	150	750	520

^a^Weight in %.

^b^MPN/g ds.

### Physico-chemical and microbiological analysis of composting stage

#### pH

**Figure 1:**
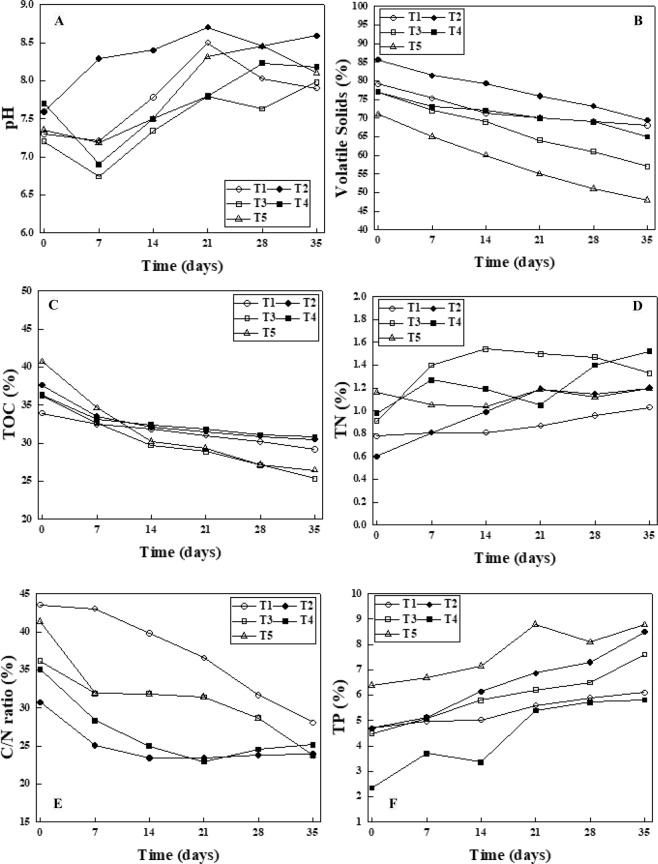
Variation of pH (**A**), TOC (**B**), VS (**C**), TN (**D**), C/N (**E**), TP (**F**) during composting stage of waste activated sludge.

An increase in pH of composting process under all experimental conditions were observed, as presented in Fig. 1(A). The maximum and minimum increased trends of pH were in reactors T2 (Initial: 7.59, Final: 8.59) and T4 (Initial: 7.20, Final: 7.98), respectively. The increasing trend during composting process corroborates with the results reported by other relevant studies^1,9,34^. An increase in the pH of final composting process are assumed to be due to excess amount of mineral nitrogen content of substrate, changes in ammonium-nitrate equilibrium^35^. According to Kruskal-Wallis statistical test and probability 95%, no significant difference was observed between mean pH of all different reactors studied in this work (p-value > 0.05).

#### Total organic carbon (TOC) and volatile solids (VS)

A significant loss of total organic carbon (TOC) and volatile solids (VS) are important indications for decomposition, mineralization and maturity of composting process. In five different reactors, there was a significant reduction of TOC (35.1%), as presented in Fig. 1(B). The maximum and minimum percentage of reduction in TOC among reactors belonged to T5 (Initial: 40.7%, Final: 26.45%) and T1 (Initial: 33.95, Final: 29.19), respectively. The decrease of TOC percentage in the present study are in line with findings reported by other researchers^2,36^. The reduction of TOC and VS are assumed to be results from loss of CO_2_ following microbial respiration during composting process. Based on results from Kruskal-Wallis statistical test, there were significant differences among different reactors (p-value < 0.05). As for VS in reactors, there was a significant reduction of VS (32.0%), as presented in Fig. 1(C). The maximum and minimum percentage of reduction in VS among reactors belonged to T5 (32%) (Initial: 71.1%, Final: 48.2%) and T1 (14%)(Initial: 79.11%, Final: 68.03%), respectively. These significant reductions in the VS values are one of considerable indications for sludge stabilization. The findings obtained associated with VS reduction in the present study were higher than other research reported by^9^ (29.1%) which examined VS after composting process.

#### Total nitrogen (TN)

A considerable increase in TN content was observed in all reactors. The increase in TN content during composting process was in range 0.03–100%. However, the maximum TN value was achieved in T4 with 1.52%. During sludge composting process, some biochemical reactions affect the TN in the process such ammonification, ammonia assimilation, nitrification and denitrification as fluctuate TN values^37^. As presented in Fig. 1(D), the highest value of TN was observed in T4, ultrasonic process increase the VS and subsequently more organic compounds are consumed by microorganism and much nitrogen in forms of ammonia and N_2_O are released depending on pH conditions^38,39^. The fact of fluctuating trend in TN is that some microorganisms depending on temperature and pH are not able to do nitrification and denitrification. The increases in TN value are comparable with finding achieved by^1^ when applied nitrogen source to improve the sewage sludge composting. According to Kruskal-Wallis statistical test, significant differences are observed among different reactors (P < 0.05).

#### C: N ratio

C: N ratio was calculated from the measured values of C and N. The C: N ratio of all reactors after a 35-day period of time were in range of 23.74–28.08. The lowest value belonged to T5, where a combination of ozonation and ultrasonics was applied to primary-stabilization of sludge. C:N ratio is an important indication for maturity of compost^11,40^ and the value below 20 is indicative of acceptable maturity of compost. The final C:N ratio of T3, T4, and T5 are lower than findings reported by other researchers^6^. However, these results are comparable with results obtained from^34^ where applied successive composting and vermicomposting for biostabilization of tannery sludge mixed with cattle dung. According to Kruskal-Wallis statistical test, a significant difference was observed among different reactors (p-value < 0.05). The fluctuating trends of all reactors are shown in Fig. 1(E).

#### Total phosphorous (TP)

Total phosphorous (TP) content of composting process were significantly increased as shown in Fig. 1(F). TP content of composting varied in range of 1.01–3.81%. The maximum increase was observed in T2, followed by T4, T5, T1, and T3. The increases in TP after composting process were supported by^6,41^. Increased TP in composting process is assumed to be the results of increases in solubolization of organic matter and release phosphorous form these by microorganism present in composting process. According to Kruskal-Wallis statistical test, a significant difference was observed among different reactor (p-value < 0.05).

#### Microbial parameters

The microbial parameters in terms of total coliform (TC) and fecal coliform (FC) are presented in Table 2. The maximum and minimum reduction for TC were found to belong T3 (83%) and T1 (40%), respectively. As for FC reduction, the maximum and minimum reduction occurred in the reactors T5 (95%) and T1 (30%).According to values reported in Table 2, it can be concluded that T3, ozonation alone as primary-stabilizing method along with composting process is capable of significant reduction in all microbiological parameters. Therefore, this process can be a suitable alternative for pathogen reduction and ensure pathogen removal not safely occurred in even vermicomposting^42^. However, the simultaneous ozonation and ultrasonics integrated with composting process is the best option in terms of microbiological parameters for composting process so as to meet the standards for agricultural uses.

**Table 2:** Microbiological parameters of resultant composting process.

Microbiological parameters	T1	T 2	T 3	T 4	T 5
**TC**	240	430	75	350	168
**FC**	35	250	36	185	23

## Conclusion

The integration ozonation and ultrasonic as primary-stabilizing methods along with composting as biological process is deliberated to be cost-effective and environmentally friendly for sludge stabilization before land and agronomic applications. A high degree of WAS stabilization was achieved in the study in terms of significant reduction in VS, TOC, TC, C/N ratio and pathogens along with the substantial increase in availability of nutrients like N and P. The efficiency of ozonation process for sludge pathogens removal was considerably high compared to ultrasonic process. However, ultrasonic process is a promising technology to increase organic matters and accordingly sludge dewaterability. The physicochemical characteristics of composting results including VS, TOC, TN, C/N, and TP are in range recommended for discharge into environment so that these parameters are comparable with resultant products from vermicomposting process. Therefore, composting system integrated with ultrasonic and ozonation technologies as sludge primary-stabilization can potentially stabilizes and converts the hazardous WAS into non-hazardous nutrients-rich organic fertilizer and/or soil conditioner. It is important to note that the causes of variation of sludge characteristics following using ozonation and ultrasonic before and during composting process need further studies and consideration.

## Methods and Materials

### Waste activated sludge (WAS)

Raw waste activated sludge was obtained on periodical basis whenever required from returned activated sludge line of municipal wastewater treatment plant (MWTT), Tehran, Iran. The fresh sludge with high moisture content (MC) was collected in large-sized plastic containers and then immediately transferred to the laboratory. In order to avoid any damage to high value of MC, the samples were stored at 4 °C until further processing. The physico-chemical and microbiological characteristics of raw WAS were analyzed according to the procedures outlined in the Standard Methods^43^, as summarized in Table 1.

### Green wastes

Green wastes (i.e. fruits and vegetables and etc.) were procured from wastes produced through domestic activities as bulking materials in the composting process. The composting material were cut into 2–3 cm length pieces. Physico-chemical and microbiological characteristics of green wastes are also reported in Table 1.

### Ozonation/Ultrasonic for sludge primary-stabilization

**Figure 2:**
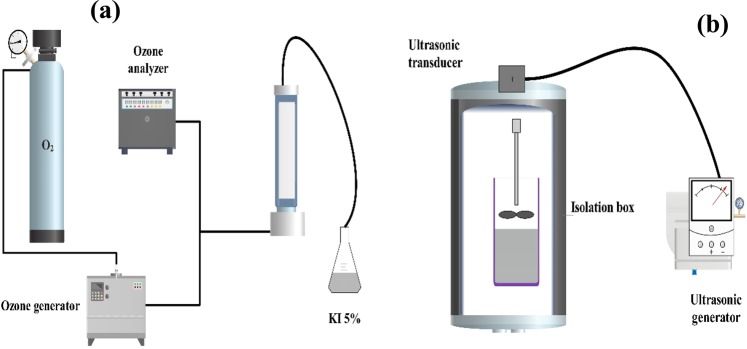
Schematic reactors: Ozonation (**a**) and Ultrasonic (**b**) for sludge semi-stabilization (Designed by Adobe Illustrator software (Version 2019) https://www.adobe.com/products/illustrator.html.

Sludge ozonation was conducted in a 3-L volume batch reactor made of polymethyl methacrylate at temperature room)25 °C). Ozone was generated from pure oxygen by an ozone generator (Denali Pasargad, Iran). The oxygen gas flow rate was 1.5 L/min with an ozone mass concentration of approximately 6 g/L. Ozone was distributed into sludge sample by a microporous diffuser at the bottom of the reactor. Ozone consumption (mg/L) was calculated based on the amount of ozone feed to reactor minus amount leaving reactor. Amount of extra ozone leaving the reactor was measured by iodometric method. Figure 2(a) shows a schematic of ozonation reactor used in the present study. After ozonation, the physico-chemical and microbiological properties of resultant sludge were characterized, as presented in Table 1.

Floc deagglomeration experiment was performed using an ultrasonic homogenizer (Bandling sonoplus HD 2070, Germany) with an operating frequency of 20 kHz and power inputted to sludge was 120 W. The ultrasonic transducer was equipped with a titanium alloy horn with a 6-mm diameter. The ultrasonic irradiation time was 1 min, which was determined by the preliminary experiments. The schematic ultrasonic reactor is presented in Fig. 2(b). As well, the physico-chemical and microbiological properties of sludge after ultrasonic treatment are summarized in Table 1.

### Experimental design

Five rectangular plastic containers with size of 0.3 m (length) × 0.3 m (width) × 0.2 m (height) were filled with a ratio of 2:1 green wastes to sludge (weight basis)^9^. Three kilograms of feed mixture was put in each rectangular plastic reactor. Composting experiments were established for 35 days and the sampling were prepared with a 7-day interval on days 0, 7,14,21,28 and 35. The zero day refers to the time of initial mixing of the sludge with different properties and green wastes before preliminary decomposition. The samples were air dried and ground in a stainless steel blender and stored in plastic vials for further chemical analysis. The experiments of each parameter was conducted in triplicate under laboratory conditions and the value reported on average. In order to maintain the temperature, eliminate volatile toxic substances and ensure aeration, each heap pile was turned over manually every 24 h throughout the study period. Heap pile temperature was measured at two-third depth from heap surface. The pH was measured by a pH meter. The composition of sludge and green wastes in five different reactors are summarized in Table 3.

**Table 3:** Composition of Treatments.

Treatment	Treatment Description
**T**_**1**_	Composting material (control)
**T**_**2**_	Raw sludge + composting material
**T**_**3**_	Ozonized sludge + composting material
**T**_**4**_	Ultrasonic sludge + composting material
**T**_**5**_	Combined ultrasonic/ Ozonized sludge + composting material

### Physico-chemical and microbiological analysis

Compost were tested for evaluation the parameters affecting sludge stabilization. The samples were prepared on days 0, 7, 14, 21, 28, and 35 in triplicate and the values of parameters were reported on average. A sample of each treatment was collected and dried at 105 °C for 1 hour and then ground for estimation of various parameters except pH, moisture content (MC). The pH was measured using digital pH meter (HQ40d, HACH, USA) in 1/10 (w/v) of composting sample to deionized water. Moisture content was determined after drying the sample at 70 ± 2 °C for 24 h using hot air oven. Total solids (TS) were determined with the difference between before and after drying the sample at 105 °C. The VS content was determined by combusting the dried sample at 550 °C for 2 h in muffle furnace. Total organic carbon (TOC) was measured on dried samples using total organic carbon analyzer (TOC analyzer, multi N/C 3100, Germany). Total nitrogen was measured by micro Kjeldahl method^44^. C:N ratio was calculated from the measured values of C and N. Total P was measured by acid digesting the samples and subsequently using ammonium molybdate method^43^. The microbiological characterization of sample including total coliform (TC) and fecal coliform (FC) were analyzed according to Multiple-tube Fermentation Technique (9221 C and 9221E, standard methods of water and wastewater examination). All samples were measured in triplicate.

### Statistical analysis

The data obtained from experiment were analyzed statistically using Minitab 16.0 software and GraphPad 5. The non-parametric statistical analysis Kruskal-Wallis was conducted at the 95% confidence level (significance was defined as P < 0.05) to analyze the significant difference between different experimental conditions (Parameters studied) in five reactors studied for different chemical parameters.
